# Synergy of Sodium Nitroprusside and Nitrate in Inhibiting the Activity of Sulfate Reducing Bacteria in Oil-Containing Bioreactors

**DOI:** 10.3389/fmicb.2018.00981

**Published:** 2018-05-16

**Authors:** Tekle T. Fida, Johanna Voordouw, Maryam Ataeian, Manuel Kleiner, Gloria Okpala, Jaspreet Mand, Gerrit Voordouw

**Affiliations:** ^1^Petroleum Microbiology Research Group, Department of Biological Sciences, University of Calgary, Calgary, AB, Canada; ^2^Department of Geosciences, University of Calgary, Calgary, AB, Canada; ^3^Department of Plant and Microbial Biology, North Carolina State University, Raleigh, NC, United States

**Keywords:** souring control, biocide, sulfate reduction, hydrogen sulfide, sodium nitroprusside

## Abstract

Sodium nitroprusside (SNP) disrupts microbial biofilms through the release of nitric oxide (NO). The actions of SNP on bacteria have been mostly limited to the genera *Pseudomonas*, *Clostridium*, and *Bacillus*. There are no reports of its biocidal action on sulfate-reducing bacteria (SRB), which couple the reduction of sulfate to sulfide with the oxidation of organic electron donors. Here, we report the inhibition and kill of SRB by low SNP concentrations [0.05 mM (15 ppm)] depending on biomass concentration. Chemical reaction of SNP with sulfide did not compromise its efficacy. SNP was more effective than five biocides commonly used to control SRB. Souring, the SRB activity in oil reservoirs, is often controlled by injection of nitrate. Control of SRB-mediated souring in oil-containing bioreactors was inhibited by 4 mM (340 ppm) of sodium nitrate, but required only 0.05 mM (15 ppm) of SNP. Interestingly, nitrate and SNP were found to be highly synergistic with 0.003 mM (1 ppm) of SNP and 1 mM (85 ppm) of sodium nitrate being sufficient in inhibiting souring. Hence, using SNP as an additive may greatly increase the efficacy of nitrate injection in oil reservoirs.

## Introduction

Sodium nitroprusside (Na_2_[Fe(CN)_5_NO]. 2H_2_O; SNP) is used as a low cost, effective, fast-acting and safe vasodilating drug for treatment of pulmonary hypertension (Koslyk et al., 2015; Lim and Zaphiriou, 2016). Although it has not been used as a biocide, the bactericidal or biofilm dispersal effect of SNP has been reported for *Pseudomonas aeruginosa* (Barraud et al., 2006; Barnes et al., 2013), *Clostridium sporogenes* (Joannou et al., 1998), and *Bacillus subtilis* (Moore et al., 2004). The bactericidal effect of SNP is suggested to be due to the release of highly reactive nitric oxide (NO) (Barraud et al., 2006; Chua et al., 2014). NO can be oxidized or reduced to give highly reactive compounds known as reactive nitrogen species (RNS). RNS are known to react with cellular components such as thiols, lipids and metals, resulting in inhibition of cellular metabolism and damage to membranes and DNA (Rogstam et al., 2007). In *P. aeruginosa*, NO release from SNP has been implicated in cell lysis and biofilm dispersal (Barraud et al., 2006; Barnes et al., 2013) suggesting that it can be used to inhibit biofilm formation in pipelines or on reservoir rocks. SNP was reported to react with H_2_S synthesized in mammals for the biosynthesis of sulfur-containing amino acids and other metabolites (Quiroga et al., 2011).

Although medical and biofilm dispersal applications of SNP are well known, its application as a metabolic inhibitor of sulfate-reducing bacteria (SRB) and as an agent for removal of sulfide for industrial applications has not been documented. SRB anaerobically respire sulfate to sulfide (H_2_S and HS^-^ at neutral pH) as part of their energy metabolism. This process, referred to as souring, is a common problem in the petroleum industry in reservoirs, in surface processing facilities of oil production operations, and in municipal wastewater systems under low- and high- temperatures as well as saline or fresh water conditions. Souring must be controlled due to the increased risk to health and safety, deterioration of oil and gas quality, and biocorrosion of pipelines and steel infrastructure (Hubert et al., 2005; Tang et al., 2009). The annual global cost of corrosion alone was estimated to be 2.6 trillion US dollars in 2016 (Koch et al., 2016). Souring is commonly controlled by the application of biocides or nitrate (Bødtker et al., 2008; Xue and Voordouw, 2015). Biocides, such as benzalkonium chloride (BAC), glutaraldehyde (Glut), bronopol, formaldehyde, cocodiamine (Coco), and tetrakis(hydroxymethyl)phosphonium sulfate (THPS) are organic chemicals commonly applied to injection waters and production facilities to kill SRB (Xue and Voordouw, 2015). Nitrate is often injected to control reservoir souring and enriches nitrate-reducing bacteria (NRB), which inhibit SRB by outcompeting for electron donors, increasing the redox potential, or producing nitrite which is an inhibitor of dissimilatory sulfite reductase (Dsr), responsible for reduction of sulfite to sulfide (Bødtker et al., 2008; Voordouw et al., 2009; Xue and Voordouw, 2015).

The application of biocides has several drawbacks including environmental concerns such as non-target toxicity and economics of application (Whitham and Gilbert, 1993; Telang et al., 1998; Fraise, 2002). Other problems are the need for high concentrations (slugs) and frequent (e.g., weekly) treatments, especially when dealing with biofilms (Kjellerup et al., 2005). Moreover, biocide performance may be constrained by poor permeability, sorption to reservoir minerals, and reaction with sulfide in reservoirs in a manner, which compromises efficacy (Reinsel et al., 1996; Gardner and Stewart, 2002; Kjellerup et al., 2005). Therefore, the choice of biocide and the optimal dosing strategy are important in the oil industry to maximize efficient control of souring. Here, we report the use of SNP as a new and effective biocide toward SRB, which is highly synergistic with nitrate.

## Materials and Methods

### Determination of Minimum Inhibitory Concentration of SNP at Low Temperature

The mesophilic SRB (mSRB) enrichment used was obtained from produced water samples of the Medicine Hat Glauconitic C (MHGC) oil field near Medicine Hat, AB, Canada (Fida et al., 2016). Duplicates of 20 mL Coleville synthetic brine medium A or K [CSBA or CSBK (Hubert et al., 2003; Callbeck et al., 2013)] were used in 60 mL serum bottles with a headspace of 90% N_2_ and 10% CO_2_ (N_2_-CO_2_) for enrichment and growth of SRB consortia. These media were buffered with bicarbonate (NaHCO_3_, 30 mM). One mL each of trace elements and tungstate/selenite solution (Widdel et al., 1983) were added separately. The pH was adjusted to 7.4 using 2 M HCl. The serum bottles were sealed with butyl rubber septa and tightened with aluminum crimp seals. Sodium sulfate was used as the electron acceptor and 3 mM volatile fatty acids (VFAs: a mixture of 3 mM each of acetate, propionate and butyrate), lactate, or MHGC heavy oil (API gravity of 16°) were used as electron donors for enrichment of SRB at concentrations indicated below.

The minimum inhibitory concentration of SNP on mSRB consortia in batch culture in serum bottles was determined for dilute cultures (0.4 μg mL^-1^ of protein) at the start or for dense cultures (80 μg mL^-1^ of protein) at the mid-log phase of growth. Different concentrations of SNP (0, 1, 10, 25, or 50 μM, where 10 μM = 3 ppm) were added to duplicates of 20 mL of CSBK medium containing 2 mM Na_2_SO_4_ and 3 mM of each VFA in 60 mL serum bottles closed with butyl rubber stoppers. These bottles were then inoculated with 100 μL of an mSRB consortium enriched for 96 h on CSBK medium with 3 mM VFA and 2 mM sulfate in dilute cultures. For experiments with dense cultures, mid-log phase cultures of mSRB enrichment were grown in duplicates in 350 mL of CSBK medium with 15 mM of each VFA and 10 mM sulfate in 500 mL bottles closed with butyl rubber stoppers and sealed with open top bottle caps. After reduction of 5 mM sulfate (i.e., in mid-log phase; about 80 μg mL^-1^ of protein) 20 mL of the cultures and 10 mL headspace gas were transferred with a syringe to 60 mL serum bottles closed with butyl rubber stoppers and filled with N_2_-CO_2_. The selected concentrations of SNP (0, 0.01, 0.025, 0.05, 0.1, 0.5 or 1 mM; 1 mM = 300 ppm) were then immediately added. The experiments were done in duplicates for each concentration and the bottles were incubated at 30°C in the dark. Samples (0.5 mL) were transferred to a microfuge tube and centrifuged at 17,000 × *g* for 5 min. Concentrations of sulfate, nitrate and nitrite were periodically determined from the clarified fluid with high-performance liquid chromatography (HPLC), using a Waters 1515 HPLC instrument equipped with a Waters 2489 UV/visible detector (for nitrate and nitrite) (Fida et al., 2016) or a Waters 432 conductivity detector (for sulfate) as described elsewhere (Grigoryan et al., 2008). The SNP concentration was determined using a Waters 1515 HPLC instrument equipped with a Waters 2489 UV/visible detector and a Nova-PAK C_18_, 3.9 × 150-mm column (Waters, Japan). Methanol:water (95:5%) was used as the mobile phase for detection of SNP. Samples (100 μL) were diluted in 400 μL of the mobile phase and injected (50 μL) at a flow rate of 1 mL min^-1^. The peak of SNP was detected at 210 nm and concentrations were estimated from known concentrations of SNP standards prepared in CSBK medium. The aqueous sulfide concentration was determined colorimetrically with *N,N*-dimethyl-*p* phenylenediamine reagent (Trüper and Schlegel, 1964).

### Determination of Minimum Inhibitory Concentration of SNP at High Temperature and Salinity

Experiments similar to those conducted with mSRB were also used to determine the efficacy of SNP at high temperature and seawater salinity. Thermophilic SRB (tSRB) were obtained as an enrichment culture of produced water samples of the Terra Nova oil field in Canada. This oil field has a resident temperature of 95°C and is located offshore of Newfoundland, Canada (Okpala et al., 2017). The samples were enriched at 60°C in CSBA medium containing 0.5 M NaCl, 10 mM sulfate and 20 mM lactate. The tSRB were grown in 400 mL CSBA medium with 20 mM lactate and 8 mM sulfate in a 500 mL bottle closed with a butyl rubber stopper and sealed with an open top bottle cap. After reduction of 4 mM sulfate, duplicate volumes of 20 mL of the culture were transferred with a syringe to 60 mL serum bottles closed with butyl rubber stoppers and filled with N_2_-CO_2_. SNP (either 0, 0.025, 0.05, 0.1, 0.5, or 1 mM) was added immediately. Samples were incubated at 60°C and sulfate reduction was monitored over time.

### Souring Control in Bioreactors Containing Heavy Oil

Glass barrel columns (500 mL) were provided with a layer of glass wool and a layer of polymeric mesh and were then packed tightly with sand (Sigma-Aldrich, 50–70 mesh), followed by a top layer of glass wool as described elsewhere (Kryachko and Voordouw, 2014; Gassara et al., 2015; Fida et al., 2017). The typical column had six sampling ports sealed with rubber stoppers and aluminum rings, allowing sampling at different positions along the flow path (Supplementary Figure S1). A large stopper perforated with a syringe needle was used to seal the column outlet. The columns were weighed before and after saturation with CSBK to measure the pore volumes (PV: typically 120 mL) and flooded with 1 PV of MHGC heavy oil replacing about 0.95 PV of CSBK. Oil was then produced from the columns by injection of anoxic CSBK medium containing 2 mM sulfate from a 2 L medium reservoir at a rate of 0.5 PV per day until the column contained 0.5 PV of residual oil with a constant supply of N_2_-CO_2_ in the headspace (Fida et al., 2017). The columns were then injected with 1 PV of mSRB consortia enriched with CSBK with 5 mM sulfate and MHGC oil. The columns were incubated for 1 month to allow cell proliferation, followed by a continuous flooding of CSBK with 2 mM sulfate (CSBK-S) at a rate of 0.25 PV per day. After establishment of souring (effluent sulfide concentrations approaching 2 mM), the medium was amended and flooded with 2 PV each of 1, 2, or 4 mM of nitrate or with 0, 0.003, or 0.015 mM of SNP. To determine the synergy between nitrate and SNP in controlling mSRB activity, the medium was amended subsequently with 0.5, 1, or 2 mM of nitrate combined with 0.0003 mM of SNP, flooding each time with 2 PV. The mSRB inhibitory activity was compared with the medium containing nitrate without SNP. Sulfide and sulfate concentrations were then determined by sampling 300 μL of fluids from the effluent and from the different ports using syringe needles. Duplicate bioreactors were used for each condition.

### Effect of SNP on the MPNs of SRB and Acid-Producing Bacteria (APB)

An anaerobic enrichment of mSRB, which also contained APB, was grown in CSBK medium with 4 mM lactate and 2 mM sulfate. Sub-cultures (10 mL) were transferred to duplicate 60 mL serum bottles closed with butyl rubber stoppers and SNP (0.5 mM) was added. Controls without SNP were incubated under the same conditions. After 10 min, 1 h and 24 h, aliquots of 100 μL of all cultures were serially diluted in triplicates in 48-well microtiter plates containing 900 μL of Postgate medium B (Shen and Voordouw, 2015) for SRB or phenol red broth (Difco, BD) for APB. The samples were incubated anaerobically for 3 weeks at 30°C in the hood_._ Wells exhibiting a black precipitate or a yellow color were scored positive for growth of SRB or APB, respectively. Most probable numbers (MPNs) were derived from the data using appropriate statistical tables (Shen and Voordouw, 2015).

### Comparison of the Effectiveness of SNP With Other Biocides

Different concentrations (5, 10, 20, or 40 ppm) of SNP or other biocides were compared. Other biocides included BAC, Glut, BAC_Glut (a combination of BAC and Glut), THPS and cocodiamine (Coco). The experiments were conducted in triplicates of 19 mL CSBK medium containing 20 mM sulfate and 10 mM lactate in 25 mL Hungate tubes inoculated with 1 mL of SRB consortia. After 1 month of incubation at room temperature, the concentrations of sulfate, sulfide, and lactate were determined.

### Proteomic Response of SRB to SNP

To elucidate the proteomic response of SRB to SNP, *Desulfovibrio vulgaris* Hildenborough was selected as a model organism. The strain was grown in four 60 mL serum bottles containing 20 mL of Postgate medium C (Nemati et al., 2001; Greene et al., 2006) with an N_2_-CO_2_ headspace at 30°C. At the mid-log phase of growth (300 μg mL^-1^ of protein), the cultures were divided into two (10 mL) and transferred to serum bottles filled with N_2_-CO_2_. SNP (0.25 mM) was added to half of the bottles whereas the other halves were kept as controls. After 3 h of exposure, 1 mL of the cultures was pelleted by centrifugation at 10,000 × *g* for 5 min and kept at -80°C until processing. Detailed methodologies for protein extraction, sample processing and proteomic data analysis are described in the Supplementary Materials. The data presented are averages of four biological replicates, which were each subjected to mass spectrometric analyses. The mass spectrometry proteomics data and the protein sequence database have been deposited to the ProteomeXchange Consortium (Vizcaino et al., 2014) via the PRIDE partner repository with the dataset identifier PXD007623.

## Results and Discussion

### Effect of SNP on Batch Cultures of mSRB

**FIGURE 1:**
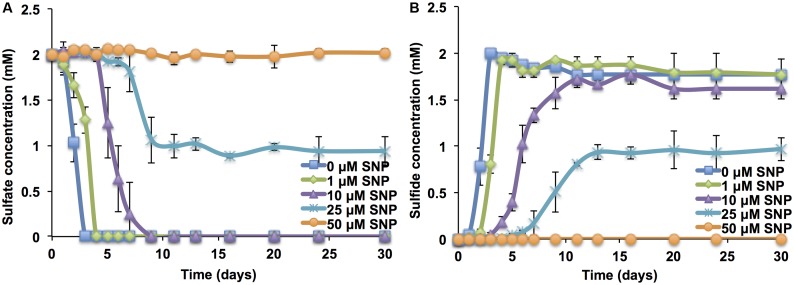
Sulfate consumption **(A)** and sulfide production **(B)** by batch cultures of mSRB in CSBK medium containing 2 mM sulfate and 3 mM VFA at 30°C in the presence of different concentrations of SNP added at the start of cultivation (*t* = 0 days); 50 μM SNP is 15 ppm.

When SNP was added to the mSRB enrichment at the start of growth, very low concentrations (less than 0.025 mM; 7.5 ppm) were required to inhibit the activity of mSRB. Sulfate consumption or sulfide accumulation was incomplete at 0.025 mM and was not observed at and above 0.05 mM (15 ppm) (**Figures 1A,B**). This suggested that 0.05 mM (15 ppm) of SNP is required to inhibit mSRB activity when added to dilute cultures (0.4 μg mL^-1^ of protein). SNP concentrations as low as 0.025 mM have been shown to decrease biofilm formation in *P. aeruginosa* (Barnes et al., 2013). An SNP dosage of 10 ppm completely inhibited the growth of mSRB in lactate-sulfate medium as judged by a low sulfide and high sulfate and lactate concentrations (Supplementary Figures S2A–C). The other biocides tested (BAC, Glut, BAC_Glut, THPS, and Coco) were not effective at this low dosage (Supplementary Figure S2). Hence, SNP appeared superior to other biocides under laboratory test conditions.

**FIGURE 2:**
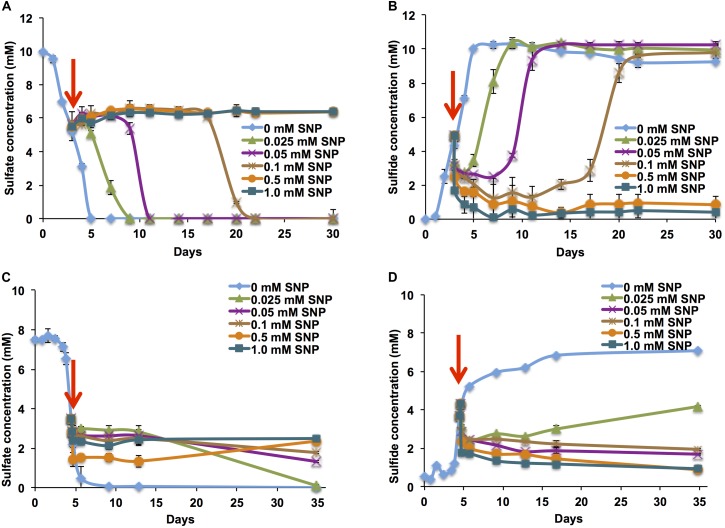
Sulfate consumption and sulfide production by mSRB and tSRB consortia grown at 30 and 60°C, respectively, in the presence of SNP. Sulfate consumption by mSRB **(A)**, sulfide production by mSRB **(B)**, sulfate consumption by tSRB **(C)**, and sulfide production by tSRB **(D)** of consortia grown in minimal medium in the presence of different concentrations of SNP added to the culture at the mid-log phase of growth (↓).

When added to batch cultures of mSRB in serum bottles at mid-log phase (dense cultures with 80 μg mL^-1^ of protein), an SNP concentration of 0.1 mM (30 ppm) extended the time needed for complete sulfate consumption from 5 to 20 days. No sulfate consumption, or accumulation of sulfide was observed at SNP concentrations of 0.5 and 1.0 mM (**Figures 2A,B**). Accumulation of 0.1 and 0.2 mM nitrate was detected in mid-log phase mSRB cultures following addition of 0.5 or 1 mM SNP, respectively, whereas accumulation of 0.1 mM nitrite was detected with 1 mM SNP only (Supplementary Figure S3). The accumulated nitrite could contribute to inhibition of SRB activity through its inhibitory effect on Dsr (Bødtker et al., 2008; Voordouw et al., 2009; Xue and Voordouw, 2015). Nitrate or nitrite accumulation was not detected when a lower concentration (0.1 mM) was used. Nevertheless, sulfate consumption was inhibited until day 17 with 0.1 mM SNP (**Figures 2A,B**), indicating that inhibition of SRB was not only caused by formation of nitrite. Inhibition by SNP could also be due to release of RNS such as NO or nitrous oxide (N_2_O), which are toxic to SRB (Londry and Suflita, 1999; Carlson et al., 2015). NO regulates enzyme activities of several metabolic pathways through nitrosylation of iron complexes or cysteine residues (Foster et al., 2003; Bertrand et al., 2012).

### Concentration-Dependent Inhibition of tSRB

The inhibition of mid-log phase tSRB (57 μg mL^-1^ of protein) was observed at lower SNP concentrations than that of mid-log phase mSRB. Reduction of sulfate to sulfide was not observed at and above 0.05 mM (15 ppm) of SNP (**Figures 2C,D**), whereas this was between 0.1 and 0.5 mM for mSRB. The increased inhibition of tSRB at low concentration of SNP was possibly due to low biomass concentration of tSRB as compared to mSRB (Joannou et al., 1998) and increased inhibitory activity of nitrite, which is rarely reduced further to N_2_ at high temperature (Perigo et al., 1967; Joannou et al., 1998; Fida et al., 2016). Similar to the low temperature conditions, SNP also reacted chemically with sulfide at 60°C as suggested from the immediate decrease of sulfide concentration upon addition of SNP (**Figures 2B,D**). These results indicate that SNP inhibits and/or kills tSRB and removes sulfide already formed at high temperature conditions. This is important for high temperature oil reservoirs flooded with seawater.

### Souring Control in Oil-Containing Bioreactors

**FIGURE 3:**
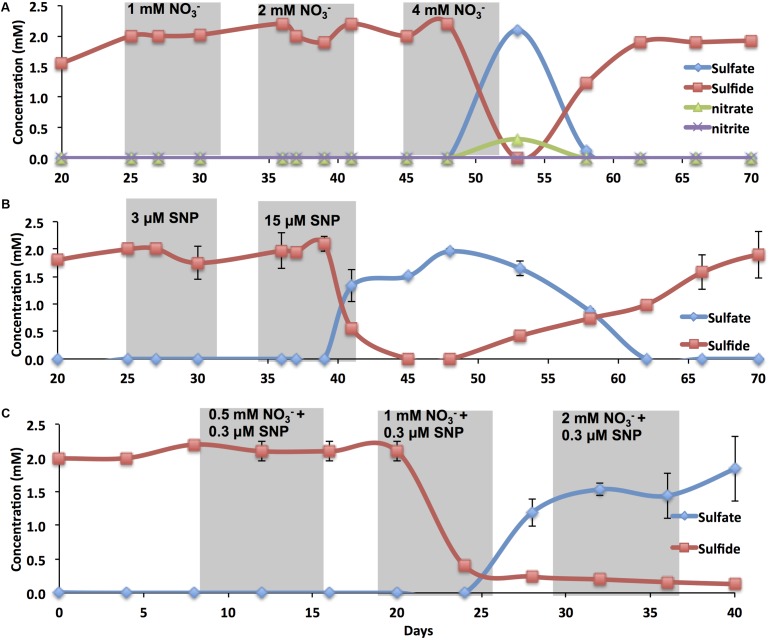
The effect of nitrate and/or SNP on sulfide production in oil-containing bioreactors. Sulfate, sulfide, nitrate, and nitrite concentrations in the effluents of oil-containing bioreactors are shown as indicated. Bioreactors were treated with nitrate **(A)**, with SNP **(B)** or with nitrate and SNP **(C)**. Shaded regions indicate the duration of treatment for injection of 2 PV (240 mL).

Sulfide and sulfate concentrations in the effluent and at the different ports were recorded during flooding of oil-containing bioreactors, which contained an active SRB consortium, with CSBK-S. Injection of 2 PV (240 mL) of CSBK-S with 1 mM (85 ppm) or 2 mM (170 ppm) of sodium nitrate did not affect the sulfide concentration in the effluent (**Figure 3A**). However, inhibition of sulfate reduction was observed with injection of 2 PV (240 mL) of 4 mM (340 ppm) of sodium nitrate (**Figure 3A**). Only partial conversion of sulfate to sulfide at lower concentrations of nitrate (1 and 2 mM) was observed at port 1, whereas at port 2 almost complete conversion of sulfate to sulfide was observed at 0, 1, and 2 mM nitrate (Supplementary Figures S4A,B). The injection of 2 PV (240 mL) of CSBK-S with 0.015 mM (4.5 ppm) of SNP resulted in souring control, which lasted long after injection of SNP was terminated at 40 days (**Figure 3B**). Sulfide concentrations decreased to zero at 45 days and sulfate concentrations reached 2 mM at 48 days after which sulfide concentrations in the effluent slowly increased again (**Figure 3B**). In the absence of SNP, partial reduction of sulfate to sulfide was observed at port 1 whereas amendment with 0.003 mM of SNP pushed sulfate reduction to port 3. Amendment with 0.015 mM completely inhibited sulfate reduction at all the ports and in the effluent (Supplementary Figures S4C,D).

A combination of 0.003 mM (1 ppm) of SNP and 1 mM (85 ppm) of nitrate, which were not efficient singly, inhibited souring at ports 1 to 6 and in the effluent of the bioreactors (**Figure 3C** and Supplementary Figures S4E,F). Inhibition was also observed with 0.003 mM of SNP and 2 mM of nitrate, but not with 0.003 mM of SNP or 0.5 mM of nitrate alone (**Figure 3C** and Supplementary Figures S4E,F). These results indicated a strong synergistic inhibition of mSRB when SNP was used in combination with nitrate. This synergistic effect allows us to minimize the nitrate dosage required to inhibit SRB, minimizing costs associated with souring control and decreasing the environmental impact from using higher doses. The results also suggest that SNP is likely not reacting with oil components. It maintained its inhibitory activities either singly or in combination with nitrate in the presence of oil. Therefore, as little as 0.003 mM (1 ppm) of SNP when combined with 1 mM (85 ppm) of sodium nitrate may effectively inhibit SRB activity in reservoirs where souring is detected.

### Chemical Reaction of SNP With Sulfide

Sodium nitroprusside not only inhibits SRB but also chemically reacts with the sulfide formed. Addition of SNP to a sour system decreased the concentration of sulfide and gave multiple reaction products with distinct colors (**Figures 2B,D** and Supplementary Figure S5). The kinetics of this chemical reaction was determined by combining 2 mM sulfide with 2 mM SNP and monitoring over a period of 2 h. A Prussian blue type color was immediately formed upon addition of SNP, which changed to a dark blue color within 1 min (Supplementary Figure S5). All the sulfides disappeared and the SNP concentration decreased from 2 to 1.2 mM within 2 h (Supplementary Figures S6A,B). Previous studies on the physiological effect of SNP as a vasodilating drug determined its possible reaction with H_2_S biosynthesized in the human body (Rock and Swinehart, 1966; Filipovic et al., 2013; Cortese-Krott et al., 2014). The blue colored product had no inhibitory effect on bacteria such as *Clostridium sporogenes* (Joannou et al., 1998). In our experiments, the dark blue color changed to dark green within 1 h and then to a brownish color over 24 h, which remained stable (Supplementary Figure S5). These changes in color indicated the occurrence of chemical reactions between SNP and sulfide, which was also evident from the disappearance of sulfide and removal of the typical H_2_S smell from the bottles.

The immediate product of reaction of SNP with H_2_S has been suggested to be the intermediate [(CN)_5_FeN(O)SH]^3-^ (Quiroga et al., 2011; Filipovic et al., 2013). However, the fate of this intermediate is not clear. Some studies suggested that the nitrososulfide ligand (HSNO) of the intermediate is further decomposed to generate NO, which is responsible for nitrosation of amines (Quiroga et al., 2011). Other studies suggest that the physiological effect of the SNP reaction product is not due to NO, which is released from HSNO only in the presence of light and under aerobic conditions, but to the formation of nitroxyl (HNO) (Filipovic et al., 2013). Filipovic et al. (2013) suggested that additional H_2_S further reacts with the HSNO to form another intermediate [(CN)_5_Fe(HNO)]^3-^ and disulfide (Filipovic et al., 2013). The disulfide is then oxidized to polysulfide which reacts with an intermediate [(CN)_5_Fe(HNO)]^3-^ to form thiocyanate and HNO with further reactions. Two HNO then react to form N_2_O and water (Filipovic et al., 2013). We detected N_2_O in the headspace of mid-log phase mSRB cultures to which 0.5 mM SNP was added (data not shown). Therefore, the mechanism of SRB inhibition by SNP in anaerobic conditions could be from the effects of NO, HNO, NO_2_, or N_2_O or other reaction intermediates.

### Effect of SNP on MPNs of SRB and APB

**FIGURE 4:**
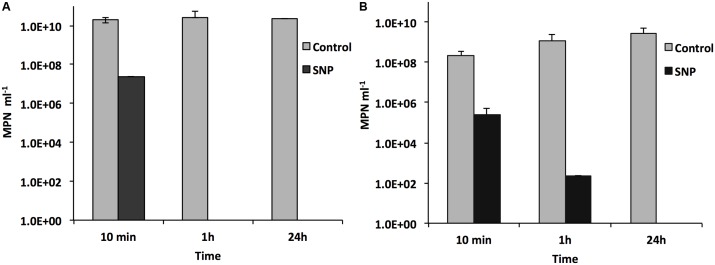
Effect of SNP on the MPNs for SRB and APB in an anaerobic consortium. The MPNs of SRB **(A)** and of APB **(B)** after 10 min, 1 h, and 24 h of exposure of an actively growing consortium to 0.5 mM SNP (black bars) are compared with the MPNs for the same consortium not exposed to SNP (gray bars).

Sodium nitroprusside decreased the MPNs of both SRB and APB. The MPN of SRB decreased by 10^3^-fold within 10 min and by 10^9^-fold or more after 1 h (**Figure 4A**). Similarly, the MPN of APB decreased by 10^3^-fold within 10 min, by 10^5^- to 10^6^-fold within 1 h and by more than 10^9^-fold after 24 h (**Figure 4B**). The results suggest that SNP killed SRB more rapidly than APB although both were completely killed after 24 h. This also suggested that SNP acts as biocide against bacteria other than SRB.

### Proteomic Response of *D. vulgaris* to SNP

**FIGURE 5:**
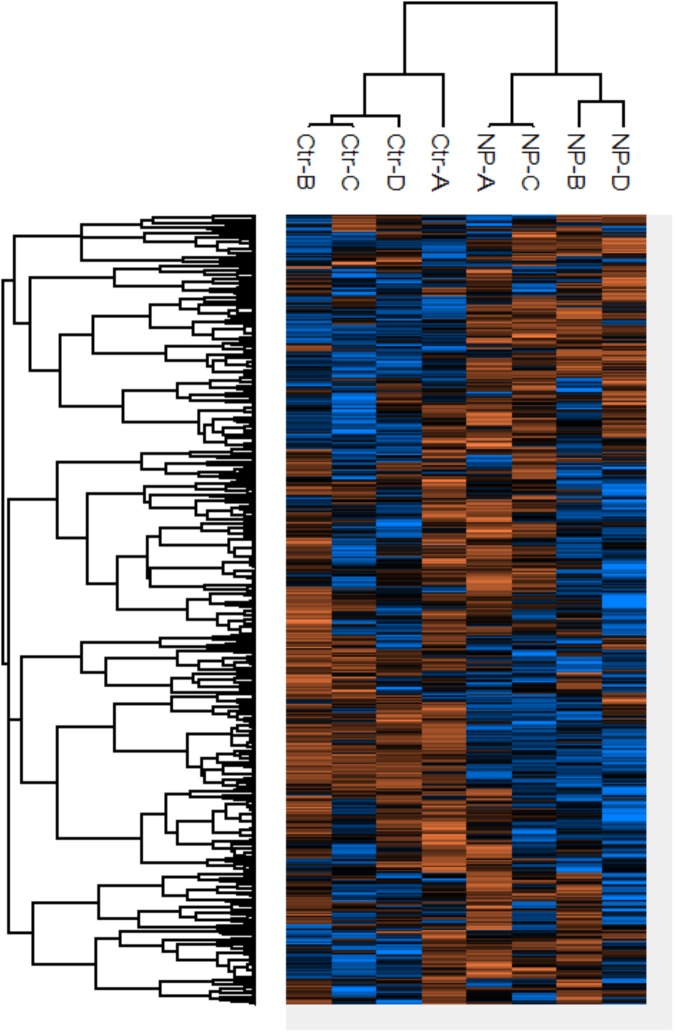
Heat map of *D. vulgaris* proteins of which the expression changed significantly between SNP treated (NP) and non-treated control (Ctr) incubations. The values were from log_2_ transformed and range from low (blue) to high (orange) abundances.

Protein expression profiles were examined for the model SRB *D. vulgaris* Hildenborough after 3 h of exposure to 0.2 mM SNP and compared to that of controls without SNP. The concentration of SNP and the exposure time were selected based on previous reports (Moore et al., 2004). The protein content decreased by three-fold within 3 h after exposure to SNP (Supplementary Figure S7). This is partly explained by the lysis of cells as reported for *C. sporogenes* (Joannou et al., 1998). Hierarchical cluster analysis of differentially expressed proteins, as depicted by the heat map, showed clustering of sample replicates into SNP treated and non-treated groups (**Figure 5**). Of the 1,127 proteins identified with a FidoCT *q*-value of <0.01, 123 proteins showed significant differences in the expression level between the control and treatment replicates (Supplementary Table S1). Of these, 49 and 74 proteins showed significantly increased and decreased expression, respectively.

Among the proteins with increased expression were those involved in stress response mechanisms. These included chaperone proteins DnaK (Q72DW8) and HtpG (Q728G0), cell division protein FtsZ (Q728V3), AhpC/Tsa family of antioxidant proteins (Q729V1), methyl-accepting chemotaxis proteins (Q72EB0 and Q727W6), an efflux transporter (Q72G03), outer membrane-associated proteins (Q72CM3, Q72D62, Q72C14, Q726K3, Q72DG2, and Q72G03), and ferrous iron transport proteins (Q728N1 and Q728N2). The increased expression of heat shock proteins is in agreement with previous findings indicating increased expression of these genes after exposure of *D. vulgaris* to different biocides (Lee et al., 2010). Heat shock proteins regulate protein homeostasis during stress conditions by facilitating the folding of nascent polypeptides and the re-folding of damaged proteins (Srinivasan et al., 2012). The increased expression of antioxidant proteins suggests that SNP-mediated stress involves oxidation of the cellular environment as previously reported for nitrous acid in *D. vulgaris* and other bacteria (Fida et al., 2012; Gao et al., 2016). SNP has been reported to induce antioxidative stress proteins involved in iron metabolism in *E. coli* (Bertrand et al., 2012) and *B. subtilis* (Moore et al., 2004).

Proteins that showed decreased expression included ribosomal proteins (Q72CH7, Q72DH2, Q72CF7, Q728R7, Q728R8, Q72CS2, Q72DQ5, and Q72CF6), periplasmic-binding amino acid transporters (Q72E67, Q72F28, Q72F29, Q72EN0, Q72FN6, Q729Q3, and Q72CP5), tryptophan synthase (Q72EU7, Q72EU8, and Q72FX8), anthranilate synthase (Q72EV0, Q72EV1, Q72EV2, and Q72EV3), and antibiotic resistance proteins such as the small multidrug resistance family (Q729L9) and the metallo-beta-lactamase family proteins (Q727Q1). Decreased expression of genes involved in ribosome activity and protein production has been reported for *D. vulgaris* exposed to free nitrous acid (Gao et al., 2016). The decreased expression of genes for resistance against antimicrobial agents suggests that SNP may increase the susceptibility of bacteria toward antibiotics. This is consistent with findings that NO increases the cell’s susceptibility to antimicrobial agents(Barraud et al., 2006; Barnes et al., 2013). Enzymes for biosynthesis of amino acids that are involved in formation of metabolites critical for survival and/or virulence and are present only in bacteria but not in humans are good candidates for anti-microbial drug discovery (Ziebart et al., 2010; Steven and Firestine, 2017). Tryptophan synthase, which was targeted by SNP, is among those candidate amino acids.

Significant differential expression of enzymes involved in energy metabolism, including sulfate reduction, such as adenosine-5′-phosphosulfate reductase (AprAB) and dissimilatory sulfite reductase (DsrAB) (Rabus et al., 2015), was not observed. Previous results also indicated no differential expression of *dsrAB* genes in *D. vulgaris* in the presence or absence of nitrate (Haveman et al., 2004). Cytochrome *c*_553_ (P04032), lactate permease (Q728C1) and periplasmic (NiFe) hydrogenase (Q06173) proteins showed significantly decreased expression. SNP also did not specifically target redox proteins involved in energy metabolism with exception of the membrane bound hydrogenases and *c*-type cytochromes involved in electron and proton transport associated with lactate-mediated sulfate reduction (Le Gall et al., 1994; Heidelberg et al., 2004).

## Conclusion

The present study identified SNP as a potent inhibitor of SRB growth and metabolic activity in both oil columns and liquid cultures. SNP was strongly synergistic with nitrate, which is commonly used to constrain SRB activity. Importantly, SNP reacts with sulfide when applied to a sour system at low concentration, which is of importance in removing sulfide and in protecting sulfide-mediated pipeline corrosion in sour reservoirs and oil processing facilities. In view of the common usage of SNP as a vasodilatory drug, no health and environmental risks are expected (Joannou et al., 1998). Because SNP has a short half-life and decays into common C, N, O, and S containing compounds, it may be regarded as a green and environmentally friendly biocide. In view of the 20-fold difference in the minimum effective dose to control souring, the use of SNP is economical compared to that of sodium nitrate. Also, because of their synergy, combinations of sodium nitrate and SNP can be price-competitive over use of either reagent alone.

## Author Contributions

TF developed the research concept, designed the experimental work, wrote the manuscript, and involved in experimental and data analysis. JV involved in experimental work of biocide comparison. TF and GO involved in biocidal activity test at high temperature. TF and JM involved in biocide experiment and data analysis. TF, MA, and MK involved in the proteomic experiment and data analysis. GV involved in developing research concept, provided funding, and reviewed and edited the manuscript.

## Conflict of Interest Statement

The authors declare that the research was conducted in the absence of any commercial or financial relationships that could be construed as a potential conflict of interest.
