# Supplementary material for: Synergy of Sodium Nitroprusside and Nitrate in Inhibiting the Activity of Sulfate Reducing Bacteria in Oil-Containing Bioreactors
Source: Front Microbiol. 2018 May 16;9:981. doi: 10.3389/fmicb.2018.00981 (PMC5965020; doi:10.3389/fmicb.2018.00981)
Supplement: Supplementary file 1 [file Data_Sheet_1.pdf]

SUPPLEMENTARY INFORMATION TO:

Synergy of Sodium Nitroprusside and Nitrate in Inhibiting the Activity of Sulfate Reducing Bacteria in Oil-Containing Bioreactors

Tekle Tafese Fida, Johanna Voordouw, Maryam Ataeian, Manuel Kleiner, Gloria Okpala, Jaspreet Mand, and Gerrit Voordouw

Sample processing and proteomic analysis

Extraction of Protein

Protein was extracted from the pellets using tryptic digest following the filter-aided sample preparation (FASP) protocol described by Wisniewski *et al.* (Wisniewski et al., 2009) with minor modifications to the protocol as described by Hamann *et al.* (Hamann et al., 2016). Protein concentrations were determined using the Pierce Micro BCA assay (Thermo Scientific Pierce, Rockford, IL, USA) following the manufacturer's instructions.

1D-LC-MS/MS

Samples were analyzed by one-dimensional LC-MS/MS using a block-randomized design (Oberg and Vitek, 2009). To reduce carry over, two wash runs and one blank run were programmed between samples. For each run, 1200 ng of extracted peptides were loaded onto a 5 mm, 300 µm ID C18 Acclaim® PepMap100 pre-column (Thermo Fisher Scientific) using an UltiMate™ 3000 RSLCnano Liquid Chromatograph (Thermo Fisher Scientific) and desalted on the pre-column. Peptides were then transferred to a 50 cm x 75 µm analytical EASY-Spray column packed with PepMap RSLC C18, 2 µm material (Thermo Fisher Scientific), which was heated to 45 °C. The analytical column was connected to a Q Exactive Plus hybrid quadrupole-Orbitrap mass spectrometer (Thermo Fisher Scientific) via an Easy-Spray source. Peptides were separated on the analytical column at a flow rate of 225 nL min^{-1} using a 260 minute gradient and mass spectra acquired in the Orbitrap as described before (Petersen et al., 2016). Around 140,000 MS/MS spectra were acquired per sample.

Protein Identification, Quantification and Statistics

A database for *D. vulgaris* Hildenborough was generated using the *D. vulgaris* proteome from Uniprot (UP000002194) containing all protein sequences derived from the genome. The cRAP protein sequence database (http://www.thegpm.org/crap/) with common laboratory contaminating proteins was added to the database. The final database contained 3634 protein sequences. The database was submitted to the PRIDE repository (see below). Protein identification was performed by searching the MS/MS spectra against the database using the Sequest HT node in Proteome Discoverer version 2.0.0.802 (Thermo Fisher Scientific) as described previously (Petersen et al., 2016). The search results for all samples were combined into a multiconsensus report using the FidoCT node in Proteome Discoverer restricting the protein-level false discovery rate (FDR) to below 5% (FidoCT q-value <0.05) (Serang et al., 2010). High confidence identifications included proteins with a FidoCT q-value of <0.01 and medium confidence identifications included proteins with a FidoCT q-value of 0.01-0.05. Based on these filtering criteria a total of 1127 proteins were identified in all samples together. Further processing was done on the multiconsensus report which was exported as a tab-delimited file.

Protein quantification was performed using Normalized Spectral Abundance Factors (NSAFs) for the proteins based on the number of peptide spectral matches (PSMs) per protein using the method described by Florens *et a* (2006). The relative abundance of a protein in a sample in % was calculated by multiplying the NSAF with 100 resulting in NSAF%.

For statistical analyses, the table with NSAFs was loaded into the Perseus software (version 1.5.6.0) (Tyanova et al., 2016). Proteins that had at least four NSAF values greater than 0 in either control or treatment samples were retained for analysis, all others were discarded. NSAF values were transformed by applying the $\log_2$. Missing values produced by $\log_2(0)$ were replaced by sampling from a normal distribution assuming that the missing values were on the lower end of abundance. A student t-test was done to detect proteins with significant difference in their expression level between control and treatment using a permutation-based FDR calculation to account for the multiple testing problems. The following parameters were used for the test: groupings

were not preserved for randomizations, both sides, 250 randomizations, FDR of 0.05 and s0 of 0.

Data Availability

The mass spectrometry proteomics data and the protein sequence database have been deposited to the ProteomeXchange Consortium (Vizcaino et al., 2014) via the PRIDE partner repository with the dataset identifier PXD007623. [Reviewer access: log in at http://www.ebi.ac.uk/pride/archive/ with username: reviewer89475@ebi.ac.uk and password: 8ZJm7Quh].

SUPPLEMENTARY FIGURES

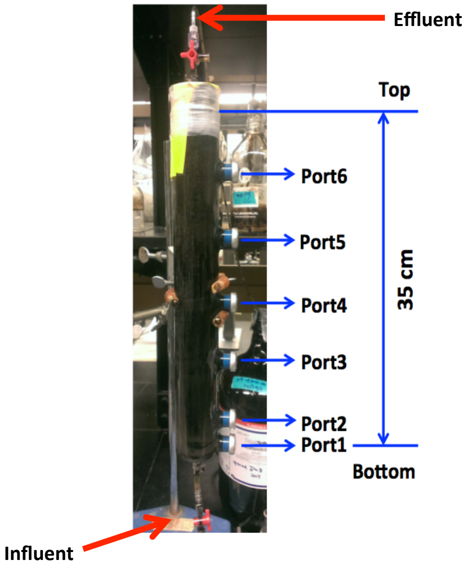

FIGURE S1 | Image of sand packed oil column showing the locations of sampling ports within the column.

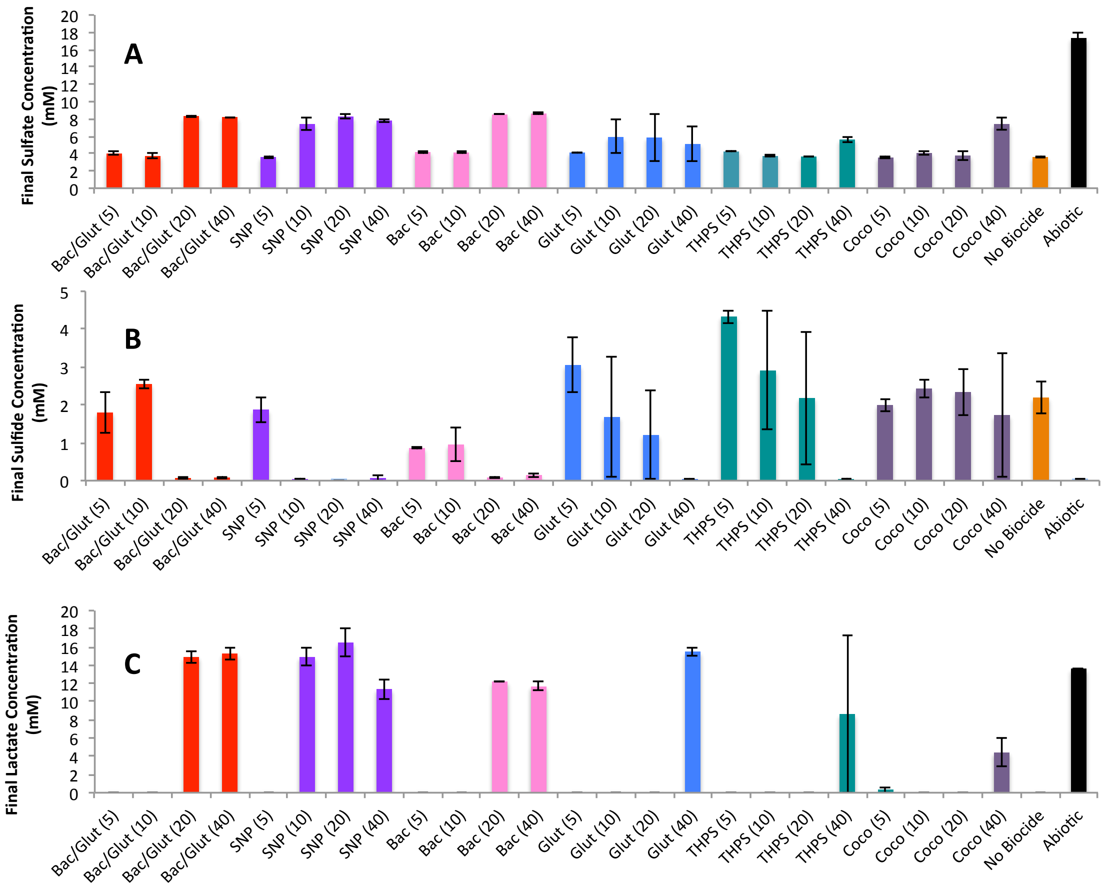

FIGURE S2 | Comparison of effect of SNP and other biocides at 5, 10, 20 or 40 ppm added at the start of growth on souring and corrosion. Concentrations of (A) sulfide, (B) sulfate, and (C) lactate were determined after one month of incubation at 23 ^{o}C with shaking in duplicate.

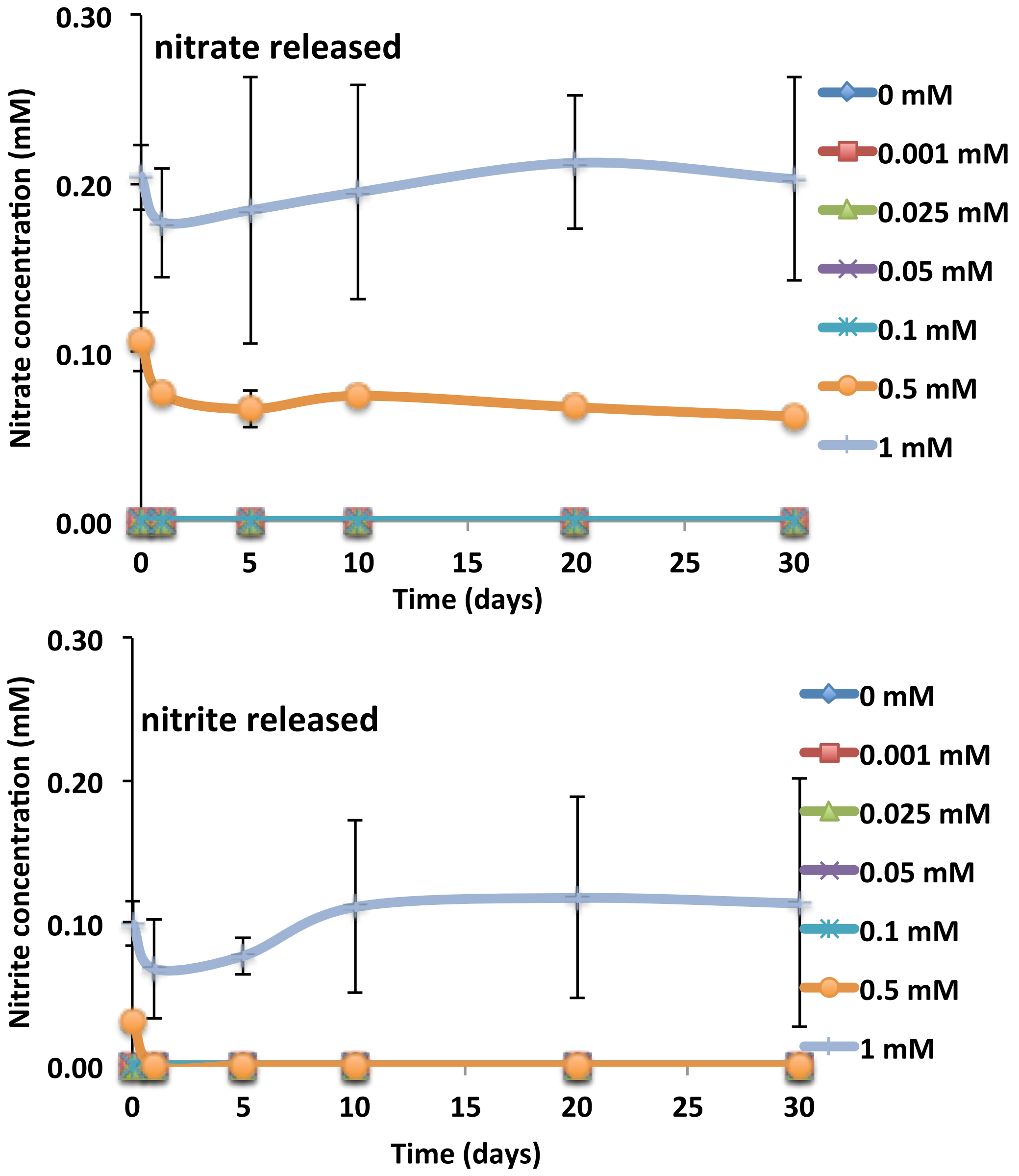

FIGURE S3 | Concentrations of nitrate and nitrite detected in mid-log phase cultures of mesophilic SRB consortia as shown in Figure 2A and 2B after addition of different concentrations of SNP.

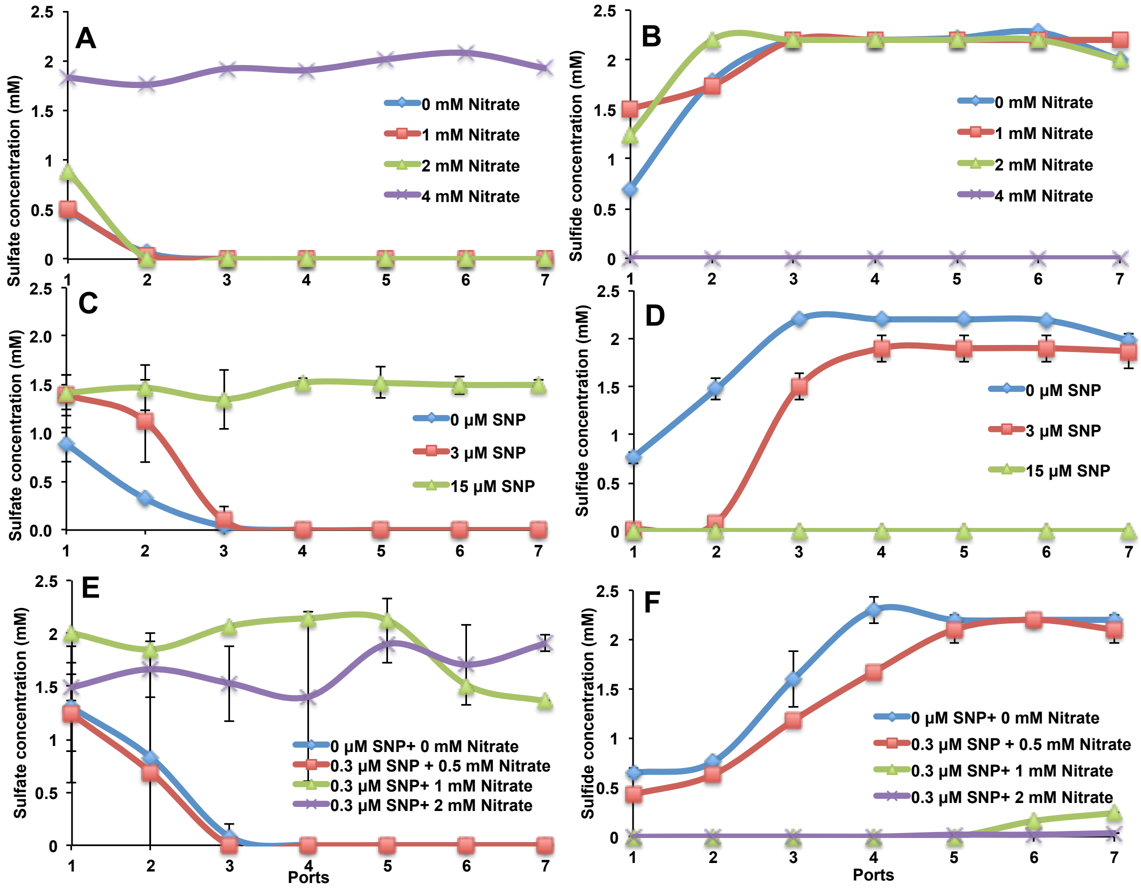

FIGURE S4 | Sulfate and sulfide concentrations at the different ports (x-axes) of the oil-containing bioreactors. Sulfate concentration in bioreactors treated with nitrate (A), sulfide concentration in bioreactors treated with nitrate (B), sulfate concentration in bioreactors treated with SNP (C), sulfide concentration in bioreactors treated with SNP (D), sulfate concentration in bioreactors treated with SNP and nitrate combination (E), sulfide concentration in bioreactors treated with SNP and nitrate combination (F). Port 7 is the effluent of the bioreactors. Samples were obtained from the ports after each treatment with two pore volumes (PV) of SNP, nitrate or SNP and nitrate.

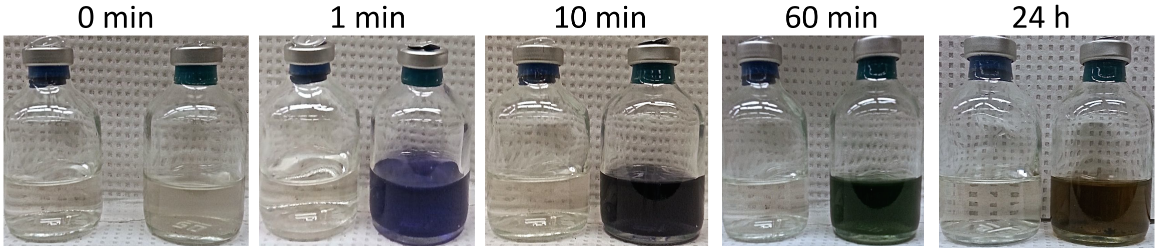

FIGURE S5 | Time-dependent change in color of serum bottles, containing CSBK medium with 0 mM (left) or 2 mM (right) of sulfide from the activity of mSRB. SNP (0.5 mM) was added to both bottles and colour development was monitored as a function of time.

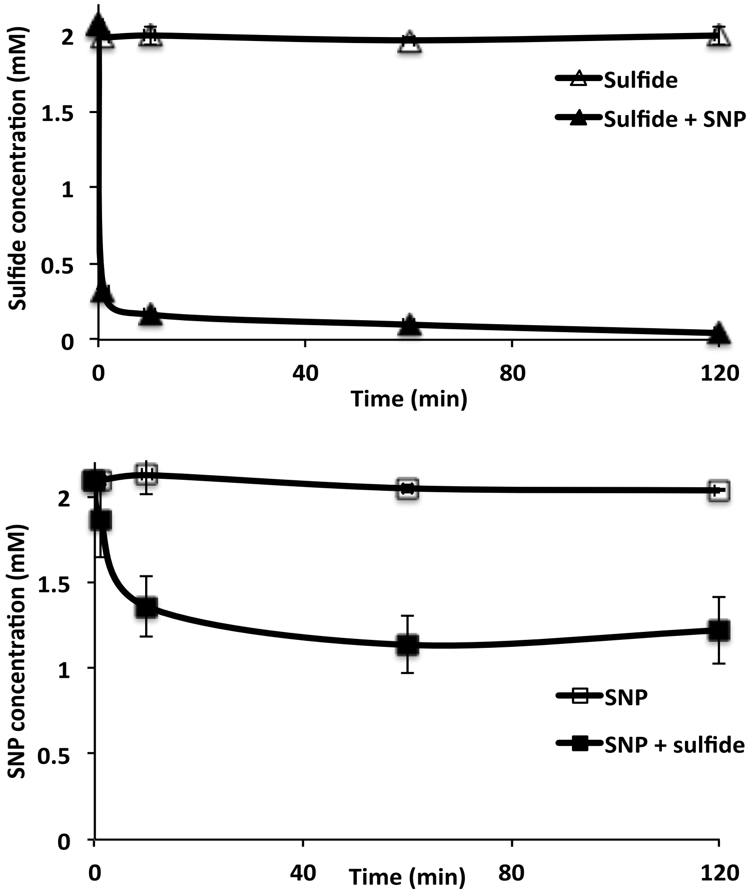

FIGURE S6 | Change in sulfide concentration (A) and SNP concentration (B) after reaction of 2 mM of SNP with 2 mM of sulfide. Controls were 2 mM sulfide without SNP (A) or 2 mM SNP without sulfide (B).

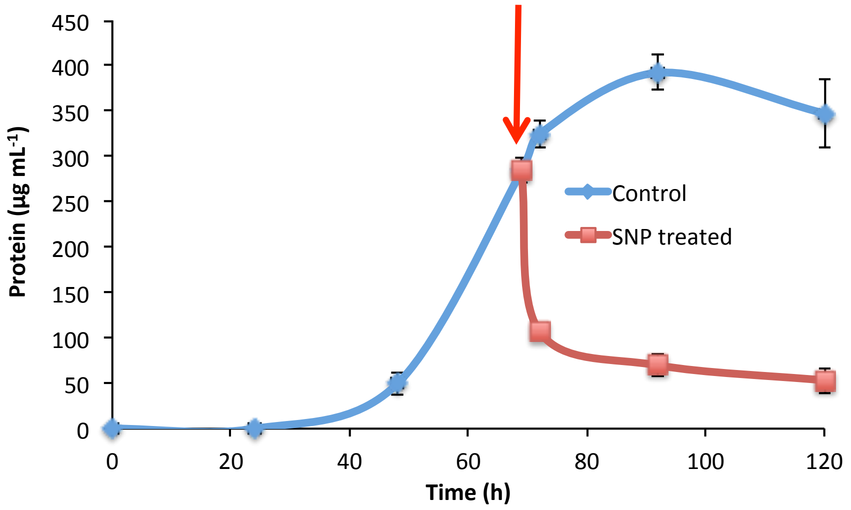

FIGURE S7 | Growth of *Desulfovibrio vulgaris* Hildenborough in Postgate medium C. SNP was added to the culture at the mid log phase of growth (↓).

TABLE S1 | Significantly expressed proteins of *Desulfovibrio vulgaris* Hildenborough in the presence or absence of sodium nitroprusside (0.25 mM)

Accession	Description	Average expression in SNP	Average expression in control
Q72CM3	Outer membrane protein P1, putative	0.06	0.03
Q728F1	Uncharacterized protein	0.02	0.01
Q72CK2	Uncharacterized protein	0.02	0.01
Q72FE1	Uncharacterized protein	0.02	0.01
Q72A92	Uncharacterized protein	0.09	0.05
Q725Z3	Uncharacterized protein	0.06	0.04
Q728N2	Ferrous iron transport protein B	0.02	0.01
P24092	High-molecular-weight cytochrome c	0.03	0.02
Q72DG2	Amino acid ABC transporter, permease protein	0.03	0.02
P33389	Protein DVU_0535	0.04	0.02
Q72DS8	Uncharacterized protein	0.24	0.17
Q72BJ9	Inorganic pyrophosphatase, manganese-dependent	1.19	0.81
Q726K3	Peptidoglycan-associated lipoprotein, putative	1.27	0.88
Q72E00	ATP synthase subunit b	0.20	0.14
Q729V1	Antioxidant, AhpC/Tsa family	0.19	0.13
Q72EB0	Methyl-accepting chemotaxis protein	0.05	0.04
Q728N1	Ferrous iron transport protein A, putative	0.05	0.03
Q726E1	Pyridine nucleotide-disulfide oxidoreductase	0.30	0.21
Q72C29	Uncharacterized protein	0.23	0.16
Q72DZ9	ATP synthase F0, B' subunit, putative	0.32	0.23
Q727U7	Cyclic dehypoxanthine futalosine synthase	0.03	0.02
Q72DY2	Uncharacterized protein	0.58	0.43
Q727W6	Methyl-accepting chemotaxis protein	0.08	0.06

Q72CH2	50S ribosomal protein L29	0.52	0.38
Q72DT1	Heterodisulfide reductase, putative	0.40	0.30
Q72EX0	GDP-mannose 4,6-dehydratase	0.10	0.07
Q726F8	Ribosomal protein S1	0.54	0.40
Q72FQ1	Carbamoyl-phosphate synthase	0.05	0.04
Q72DW8	Chaperone protein DnaK	0.33	0.25
Q728V3	Cell division protein FtsZ	0.08	0.06
Q72CH4	30S ribosomal protein S3	0.66	0.50
Q729X1	Acetyl-CoA carboxylase, biotin carboxylase, putative	0.16	0.12
Q72AR0	Adenylate kinase	0.55	0.42
Q728G0	Chaperone protein HtpG	0.08	0.07
Q727E1	Peptide chain release factor 1	0.05	0.04
Q72B07	Phosphoenolpyruvate synthase, putative	0.18	0.14
Q72E02	ATP synthase subunit alpha	0.66	0.52
Q72E04	ATP synthase subunit beta	0.73	0.58
Q72D62	Membrane protein, Bmp family	0.17	0.14
Q72C14	Peptidylprolyl isomerase	0.50	0.40
P45574	Sulfite reductase, dissimilatory-type subunit alpha	0.91	0.73
Q72CG6	30S ribosomal protein S8	0.69	0.55
P61522	Argininosuccinate synthase	0.10	0.08
Q72CG3	30S ribosomal protein S5	0.57	0.46
Q72G03	Efflux transporter, RND family, MFP subunit	0.10	0.08
Q72ER1	Translation initiation factor IF-2	0.12	0.10
Q72DT0	Heterodisulfide reductase, iron-sulfur-binding subunit, putative	0.33	0.28
Q727C7	DNA-directed RNA polymerase subunit beta	0.15	0.13
Q72C16	Peptidase/PDZ domain protein	0.10	0.09
Q72CA6	Ketol-acid reductoisomerase (NADP($^+$))	0.36	0.44
Q72AS8	Aspartokinase	0.06	0.07
Q72CH7	50S ribosomal protein L2	0.27	0.33
Q72FD1	AhpF family protein/thioredoxin reductase	0.06	0.07
Q729I6	Aminotransferase, classes I and II	0.05	0.07
Q725N2	Glutamine synthetase, type I	0.04	0.05
Q72A88	Iron-sulfur cluster carrier protein	0.25	0.32
Q72E67	Amino acid ABC transporter, periplasmic-binding	0.12	0.16
Q72DH6	Tyrosine--tRNA ligase	0.07	0.10
Q72BS5	Aldehyde oxidoreductase	0.08	0.11
Q72CQ5	Probable thiol peroxidase	0.23	0.31
Q72DH2	30S ribosomal protein S18	0.24	0.33
Q72EL3	Glyceraldehyde-3-phosphate dehydrogenase	0.06	0.08
Q72DG5	Glu/Leu/Phe/Val dehydrogenase family protein	0.02	0.02
Q72A89	MTH1175-like domain family protein	0.31	0.44
Q727S1	Dehydrogenase, FMN-dependent family	0.09	0.12
Q725I1	Glutamate-1-semialdehyde 2,1-aminomutase	0.08	0.12
Q72FG1	Oxidoreductase, FAD/iron-sulfur cluster-binding	0.07	0.10
Q729K3	Acetylornithine aminotransferase	0.02	0.03
Q72BH2	Probable GTP-binding protein EngB	0.02	0.03
Q72FH2	MTH1175-like domain family protein	0.24	0.35
Q72DQ8	Uridylate kinase	0.06	0.08

Q72DF0	DAK1 domain protein	0.07	0.11
Q726H4	Glycerol kinase	0.01	0.02
Q06173	Periplasmic [NiFe] hydrogenase small subunit 1	0.10	0.16
Q726I8	AMP-binding enzyme family protein	0.01	0.02
Q725W5	ATP-dependent RNA helicase, DEAD/DEAH family	0.07	0.10
Q72C43	Flagellin	0.02	0.03
Q72CF7	30S ribosomal protein S13	0.20	0.32
Q726Y0	Uncharacterized protein	0.01	0.01
Q72F29	Amino acid ABC transporter, periplasmic amino acid-binding protein	0.07	0.11
Q72EN0	Periplasmic branched chain amino acid-binding protein	0.19	0.31
Q72FN6	Molybdenum ABC transporter, periplasmic molybdenum-binding protein	0.09	0.15
Q728R7	50S ribosomal protein L35	0.19	0.33
Q72CS2	50S ribosomal protein L28	0.39	0.67
Q728R0	Hydroxylamine reductase	0.04	0.06
Q72AT0	CBS domain protein	0.05	0.09
Q729Q3	Glycine/betaine/L-proline ABC transporter, periplasmic-binding protein	0.02	0.03
Q72B13	Uncharacterized protein	0.08	0.14
Q72A94	Iron-sulfur cluster-binding/ATPase domain protein	0.09	0.15
Q72DQ5	30S ribosomal protein S2	0.32	0.57
P20418	Desulfoferrodoxin	0.24	0.43
P61654	2-dehydro-3-deoxyphosphooctonate aldolase	0.01	0.02
Q72EV8	2-amino-3,7-dideoxy-D-threo-hept-6-ulosonate synthase	0.24	0.45
Q72E57	Response regulator	0.00	0.01
Q728X5	Phosphate ABC transporter, periplasmic phosphate-binding protein PstS	0.01	0.02
Q726Q9	Aminotransferase, class IV	0.01	0.03
Q728R8	50S ribosomal protein L20	0.09	0.18
Q72EV6	Chorismate mutase/prephenate dehydratase	0.03	0.06
Q727E3	50S ribosomal protein L31	0.08	0.16
Q72G54	Universal stress protein family	0.01	0.03
Q725Y1	Thiamine pyrophosphate-requiring enzyme	0.01	0.02
Q72EV7	Predicted 3-dehydroquinate synthase	0.08	0.17
Q72EV5	3-phosphoshikimate 1-carboxyvinyltransferase	0.02	0.05
Q72CP5	Amino acid ABC transporter, periplasmic amino acid-binding protein	0.07	0.16
Q72CF6	30S ribosomal protein S11	0.12	0.28
Q72EZ5	Universal stress protein family	0.03	0.07
Q725Y0	Aldehyde dehydrogenase (NADP) family protein	0.01	0.02
Q725R8	NAD-dependent epimerase/dehydratase family protein	0.02	0.04
Q729L9	Smr family protein	0.01	0.02
Q72EU7	Tryptophan synthase alpha chain	0.10	0.29
Q72FX8	Tryptophan synthase beta chain	0.08	0.23
P04032	Cytochrome c-553	0.09	0.26
Q728C1	L-lactate permease family protein	0.00	0.01
Q727Q1	Metallo-beta-lactamase family protein	0.01	0.02
Q72EU8	Tryptophan synthase beta chain	0.08	0.26
Q72EV3	Anthranilate synthase, component I	0.05	0.17
Q72FH3	Uncharacterized protein	0.01	0.04
Q72F96	TPR domain protein	0.02	0.07
Q72C64	Hpt domain protein	0.01	0.02

Q72E06	Uncharacterized protein	0.03	0.13
Q72EV2	Anthranilate synthase, glutamine amidotransferase component	0.03	0.15
Q72EV1	Anthranilate phosphoribosyltransferase	0.04	0.19
Q72EV0	Indole-3-glycerol phosphate synthase	0.03	0.16

References

Florens, L., Carozza, M.J., Swanson, S.K., Fournier, M., Coleman, M.K., Workman, J.L., et al. (2006). Analyzing chromatin remodeling complexes using shotgun proteomics and normalized spectral abundance factors. *Methods* 40**,** 303-311. doi: 10.1016/j.ymeth.2006.07.028

Hamann, E., Gruber-Vodicka, H., Kleiner, M., Tegetmeyer, H.E., Riedel, D., Littmann, S., et al. (2016). Environmental Breviatea harbour mutualistic *Arcobacter* epibionts. *Nature* 534**,** 254-258. doi: 10.1038/nature18297

Oberg, A.L., and Vitek, O. (2009). Statistical design of quantitative mass spectrometry-based proteomic experiments. *J. Proteome. Res.* 8**,** 2144-2156. doi: 10.1021/pr8010099

Petersen, J.M., Kemper, A., Gruber-Vodicka, H., Cardini, U., Van Der Geest, M., Kleiner, M., et al. 2016. Chemosynthetic symbionts of marine invertebrate animals are capable of nitrogen fixation. *Nature Microbiology* [Online], 2. Available: http://dx.doi.org/10.1038/nmicrobiol.2016.195.

Serang, O., Maccoss, M.J., and Noble, W.S. (2010). Efficient marginalization to compute protein posterior probabilities from shotgun mass spectrometry data. *J. Proteome Res.* 9**,** 5346-5357. doi: 10.1021/pr100594k

Tyanova, S., Temu, T., Sinitcyn, P., Carlson, A., Hein, M.Y., Geiger, T., et al. (2016). The Perseus computational platform for comprehensive analysis of (prote)omics data. *Nat. Methods.* 13**,** 731-740. doi: 10.1038/nmeth.3901

Vizcaino, J.A., Deutsch, E.W., Wang, R., Csordas, A., Reisinger, F., Rios, D., et al. (2014). ProteomeXchange provides globally coordinated proteomics data submission and dissemination. *Nature Biotechnology* 32**,** 223-226. doi: 10.1038/nbt.2839

Wisniewski, J.R., Zougman, A., Nagaraj, N., and Mann, M. (2009). Universal sample preparation method for proteome analysis. *Nat. Methods.* 6**,** 359-362. doi: 10.1038/nmeth.1322
